# Essential medicines in conflict-affected settings: a systematic review of accessibility and affordability across fragile health systems

**DOI:** 10.1186/s13031-026-00825-x

**Published:** 2026-07-30

**Authors:** Tracy Kuo Lin, Atousa Bonyani

**Affiliations:** 1 Institute for Health & Aging, Department of Social and Behavioral Sciences, University of California San Francisco, San Francisco, USA; 2 Institute for Global Health Sciences, University of California San Francisco, San Francisco, USA

## Abstract

**Background:**

Conflict disrupts health systems and limits access to essential medicines; however, evidence on accessibility and affordability in conflict settings remains limited. This systematic review synthesizes empirical evidence on these outcomes.

**Methods:**

We conducted a systematic review following Preferred Reporting Items for Systematic Reviews and Meta-Analyses (PRISMA) guidelines. PubMed, Web of Science, Embase, and Google Scholar were searched in October 2025. Studies were eligible if they assessed accessibility to or affordability of essential medicines in conflict settings and reported quantitative outcomes. Two reviewers independently screened studies in Covidence. Risk of bias was assessed and results were synthesized using descriptive statistics and narrative analysis.

**Results:**

Fourteen studies across 12 conflict-affected settings met the inclusion criteria. Medicines for infectious diseases were generally more accessible than those for noncommunicable diseases. Public-sector facilities often reported lower accessibility than private outlets, leading patients to seek medicines in higher-priced private pharmacies and clinics. Affordability challenges were common, especially for uninsured patients and for chronic disease medicines. Key drivers included disrupted supply chains, damaged infrastructure, workforce shortages, and economic shocks such as inflation and rising transport costs.

**Conclusion:**

Conflict-affected settings face challenges maintaining equitable access to essential medicines. While medicines for acute infectious conditions are often prioritized in humanitarian responses, those for chronic diseases are less accessible and affordable. This gap may undermine continuity of care. Strengthening pharmaceutical supply chains, improving coordination between national and humanitarian procurement systems, and expanding financial protection mechanisms may improve access.

## Background

Conflict, political violence, and instability impact health systems not only directly through acute disruptions to care delivery but also indirectly by altering the political, economic, and market structures that govern access to health services and medicines [1, 2]. Over 930 million people globally are estimated to live in settings with a high propensity for repeated violent and non-violent political events that affect access to daily necessities and essential health services, including medicines. The detrimental effects of political events on health service delivery, health care utilization, and food insecurity are well documented; across conflict-affected and fragile settings, damage to infrastructure, workforce attrition, mobility restrictions, and economic volatility collectively influence how health services are delivered and sustained, shaping both supply and demand for essential medicines [3–5]. 

Essential medicines are defined by the World Health Organization (WHO) as those that satisfy the priority health care needs of the population and should always be accessible, in adequate amounts, appropriate dosage forms, assured quality, and at prices individuals and communities can afford [6]. Essential Medicine Lists (EMLs)--and at the country level, National Essential Medicines Lists (NEMLs)–represent a cornerstone of pharmaceutical policy, guiding procurement decisions, reimbursement strategies, and treatment prioritization across diverse health system contexts [7]. In conflict settings, essential medicines form the backbone of both acute and chronic care, supporting management of communicable diseases, maternal and child health conditions, trauma care, and noncommunicable diseases [8–10]. 

Reports from conflict-affected settings describe uneven patterns of medicine accessibility, in which commonly used acute-care treatments remain relatively accessible while medicines for chronic conditions become increasingly constrained, reflecting humanitarian procurement priorities, disrupted logistics, and changing economic conditions [9, 11]. When medicines are inaccessible in public facilities, patients are often forced to seek treatment in the private sector, where higher prices and out-of-pocket payments may render treatment unaffordable, further deepening health inequities during periods of conflict [12]. In many settings, conflict shifts pharmaceutical accessibility toward acute care medicines while constraining accessibility to medicines for chronic conditions. This shift has important implications for continuity of care and equitable access to treatment for chronic diseases in conflict-affected and fragile health systems [13]. These dynamics suggest that conflict may systematically affect the accessibility and affordability of essential medicines across different health system sectors.

Existing evidence on pharmaceutical accessibility in conflict-affected settings is derived largely from descriptive facility-level reports, cross-sectional audits, and short-term assessments of medicine accessibility that provide snapshots of access but offer limited insight into how repeated exposure to conflict shapes pharmaceutical pricing, affordability, and utilization over time. These studies suggest that accessibility and affordability are the two most affected dimensions of medicine access in these settings [11]. However, evidence remains fragmented across settings, health system sectors, and medicine categories. To address this gap, we conducted a systematic literature review to examine empirical evidence on the accessibility and affordability of essential medicines in conflict-affected contexts. Specifically, we aimed to synthesize quantitative findings related to medicine accessibility and affordability across different health system levels to inform policy and humanitarian response strategies. Understanding how conflict reshapes pharmaceutical access is essential for designing resilient health systems that protect both acute and chronic care in conflict-affected settings.

## Methods

This review focuses on accessibility and affordability, as these are the two dimensions of essential medicines most affected by conflict and most consistently and jointly measured in the literature [11, 14]. The WHO/HAI methodology, one of the most widely used frameworks in this field, is designed to assess both facility-level medicine accessibility and affordability simultaneously [15]. For the purposes of this review, three core concepts were defined as follows. *Accessibility* refers to the physical presence of essential medicines at the health-facility level [16]. *Affordability* refers to the ability of patients or households to purchase needed medicines without incurring financial hardship [17]. *Fragile and conflict-affected settings* are defined according to the World Bank Classification of Fragile and Conflict-Affected Situations (FCS) and comprises two lists: (1) the Public FCV List, which identifies countries where at least 20% of the population lives in areas with elevated conflict-related fatalities, and (2) the Institutional Fragility List, which identifies countries with low Country Policy and Institutional Assessment (CPIA) scores and structural governance fragility [18, 19]. These definitions guided the search strategy design, eligibility screening and data extraction throughout the review.

### Search strategy

We performed a systematic literature review following Preferred Reporting Items for Systematic Reviews and Meta-Analyses (PRISMA) guidelines to identify articles relevant to the study topic [20]. The review protocol was registered in PROSPERO (CRD420261335404). The review was restricted to the peer-reviewed studies, and gray literature was not searched. The study team searched Google Scholar, PubMed, Web of Science, and Embase in October 2025. The search strategy used context-specific keywords (e.g., conflict, violence, war) in combination with topic-related keywords associated with essential medicines. The search strategy was structured around three concept blocks combined using the Boolean operator AND: (1) essential medicines and pharmaceutical access terms, (2) access-related outcome terms including availability, affordability, supply chain, pricing, and utilization, and (3) conflict and fragility terms including specific conflict-related descriptors, humanitarian emergency terminology, displacement populations, and names of World Bank-designated fragile and conflict-affected countries. See Appendix 1 for the full search strings. No ethical approval was required, as this was a desk-based study that involved no primary data collection.

### Eligibility criteria

Articles were eligible for inclusion if they (1) described a context that is post-conflict, conflict-affected, or was impacted by war or crisis; (2) concerned national essential medicines lists or essential drug lists; (3) examined accessibility of, or affordability of essential medicines in violence-affected and conflict-affected settings; (4) reported outcomes related to accessibility and affordability of essential medicines; and (5) the article was published in English. The impact of violence and conflict on the affordability and accessibility of essential medicines may be examined at the national, regional, or institutional levels, recognizing that conflict, violence, and essential medicine-related policy implementation vary across different organizational structures and health system contexts.

Studies reporting a quantitative outcome related to accessibility and affordability were included. The review was restricted to quantitative studies as the aim was to assess the status of medicine accessibility and affordability rather than to explore the determinants, experiences, or contextual factors underlying access barriers, which qualitative approaches are better suited to address. Studies without empirical data, conference abstracts, posters, or protocols were excluded. We did not set a publication time frame and included all studies meeting eligibility criteria regardless of publication date. We followed predefined definitions of key terms when determining study eligibility. We followed the World Bank Group Classification of Fragile and Conflict-Affected Situations and included listed countries by year in our search strings to capture potentially relevant studies [18]. Nevertheless, studies were excluded if the study author(s) did not explicitly describe conflict, crisis, or war as a feature of the study context—even when the reviewers were aware that conflict had occurred in that setting.

### Risk of bias assessment

We used the Joanna Briggs Institute (JBI) Critical Appraisal Tools to assess risk of bias in the included studies. Specifically, we included the appraisal tool for analytical cross-sectional studies that contains eight criteria for the cross-sectional studies identified [21]. Since JBI does not provide an appraisal tool for narrative reviews, we used the Scale for the Assessment of Narrative Review Articles (SANRA) for quality assessment of the narrative review reporting quantitative data [22]. Studies meeting a minimum quality threshold of 75% of the total possible score were retained for inclusion. Details of our risk of bias assessment are presented in Appendix 2.

### Data extraction

We used Covidence review software (Veritas Health Innovation, Melbourne, Australia. Available at www.covidence.org*)* to manage the review process [23]. The software automatically removed duplicate records, after which the study team manually checked for remaining duplicates. For each of the identified abstracts and full texts, two reviewers (TKL and AB) independently screened studies for eligibility; disagreements were resolved through discussion. One reviewer then extracted data from eligible studies and assessed the risk of bias and was checked by the second reviewer. Data were extracted using a predefined 21-item extraction sheet in Microsoft Excel (version 16.105.3). We used R Studio (version 2025.05.1 + 513) to generate descriptive figures illustrating study findings.

### Analysis

Given the substantial heterogeneity across included studies in conflict types, settings, health system structures, and outcome measures, a formal meta-analysis or structured quantitative synthesis was not appropriate. Narrative synthesis was therefore selected to identify patterns across heterogeneous evidence.

As defined above, accessibility referred to the presence of essential medicines at the health-facility level,^16^ while affordability referred to the ability of patients to purchase medicines [24]. Reviewers summarized study results in tables by country of origin, geographical location, year of sampling, medicines assessed, health services delivery structure, accessibility and affordability outcomes (i.e., measurement and status of medicines at the facilities), and other relevant information. According to the measures reported in each study, we summarized findings related to accessibility and affordability. Descriptive analyses of key characteristics of included publications were conducted. Consensus on themes related to accessibility and affordability was reached through discussion between the two reviewers (TKL and AB). Study outcomes were synthesized using narrative synthesis by identifying patterns and themes relevant to the study question. Aggregate data and descriptive statistics such as counts, ranges, means, standard deviations, medians, and interquartile ranges, where appropriate, were presented.

### Findings

**Fig. 1:**
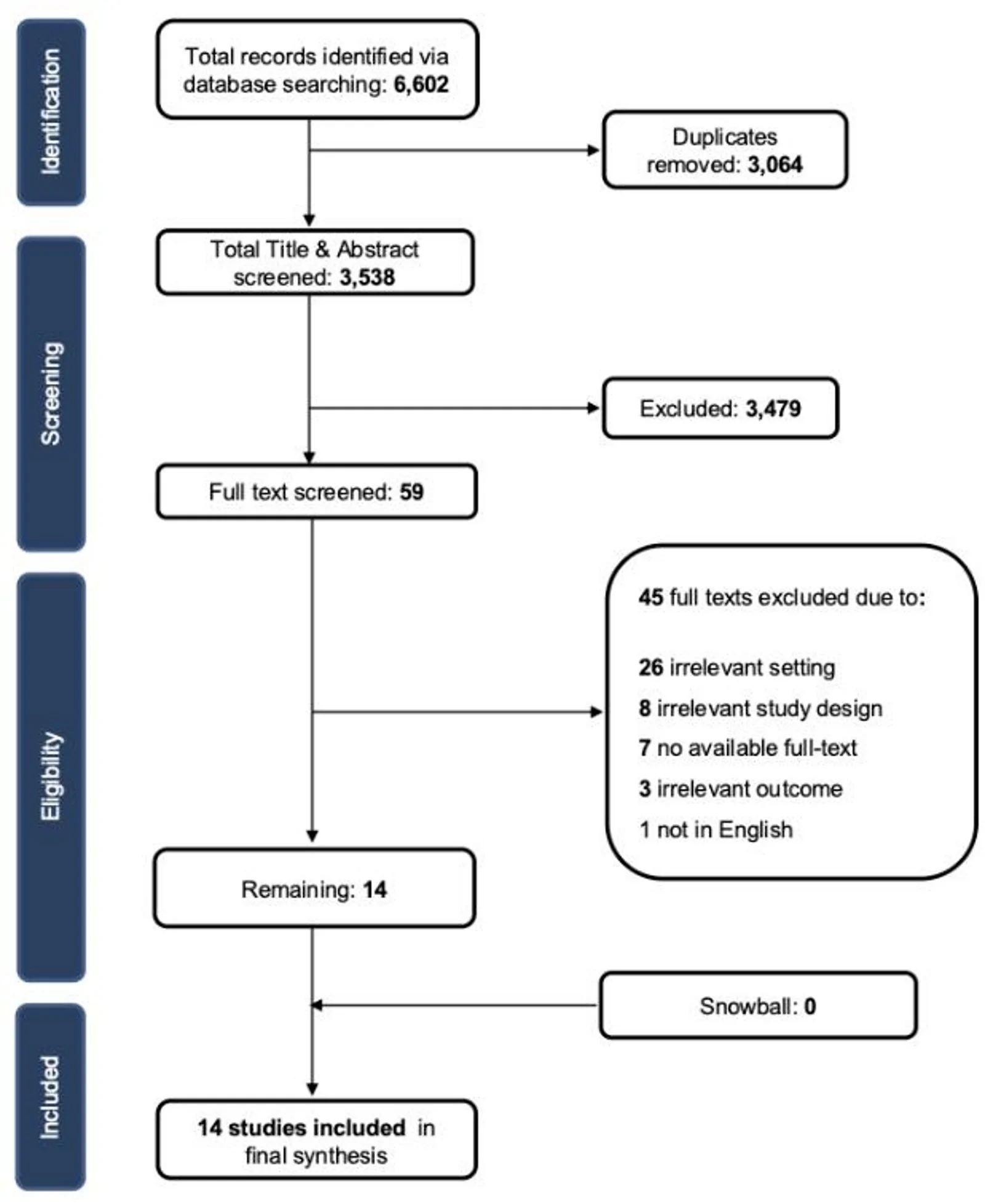
PRISMA flow diagram illustrating the study selection process

A total of 6,602 records were identified across PubMed, Google Scholar, Embase, and Web of Science. After removing 3,064 duplicates, two independent reviewers screened 3,538 titles and abstracts. Of these, 59 articles were selected for full-text review. During the full-text assessment, 45 articles were excluded because of irrelevant settings (*n* = 26), irrelevant study design (*n* = 8), no available full-text (*n* = 7), irrelevant outcome (*n* = 3), and publication in a language other than English (*n* = 1). Ultimately, 14 studies met the inclusion criteria and were included in the final analysis (Fig. 1).

The methodological quality of the included studies was assessed using design-appropriate critical appraisal tools. Thirteen analytical cross-sectional studies were appraised using the JBI Critical Appraisal Checklist for Analytical Cross-Sectional Studies, while one narrative review was assessed using the SANRA tool. All included studies met a minimum quality threshold of 75%, and none were excluded on the basis of methodological limitations. Overall, the methodological quality of the evidence base was considered high. Among the cross-sectional studies, the most common methodological limitations were the lack of explicit identification of potential confounding factors and strategies to address potential confounding. The narrative review had limitations related to the clear presentation of the literature review strategy and lack of formal uncertainty analysis. Overall, the quality appraisal indicated that the included studies provided credible evidence to address the objectives of this review.

Of the 14 studies that met the inclusion criteria, nine (64%) were from Sub-Saharan Africa, of which three focused on Sudan (23%), two on Ethiopia (14%), and South Sudan, Chad, the Democratic Republic of Congo, Uganda each represented by one study (7% each). Three studies (21%) were from the Middle East and North Africa region, covering Palestinian territory (Gaza Strip), Syria, and Yemen (7% each). One study (7%) was from Eastern Europe (Ukraine) and one (7%) from Central Asia (Afghanistan). Three studies (23%) assessed the availability of NEMLs at the national level, while the remaining 11 studies (77%) focused on specific districts. All included studies examined medicine accessibility (100%), whereas only five (36%) assessed medicine affordability. In addition, one study (7%) analyzed end-user price variations across conflict-affected areas, and one study (7%) reported patient out-of-pocket expenditures. In terms of conflict type, 13 studies (93%) were conducted in armed conflict settings, including civil war, military occupation, and blockade. The one remaining study (7%) addressed conflict caused by drought and food insecurity in north-east Uganda. Table 1 summarizes the characteristics of the included studies.

**Table 1:** Characteristics of included studies

Study	Country/setting	Year of data collection	Study setting and sample	Population	Conflict type	Accessibility measurement	Affordability measurement
van Gurp et al. [25]	Afghanistan	2021	Nationwide survey of public health facilities (885 facilities across multiple facility levels)	General population	Armed conflict and insecurity	Continuous accessibility of 31 essential medicines during the previous 30 days	Not assessed
Schäfermann et al. [26]	Cameroon; Democratic Republic of the Congo	2017–2018	Government facilities, church facilities, private pharmacies, and informal vendors (60 outlets)	General population	Armed conflict and insecurity	Percentage of facilities stocking each medicine (WHO/HAI methodology)	WHO/HAI methodology
Ragomzingba et al. [27]	Chad	2023	Public, private, religious, and pharmaceutical supply facilities nationwide	General population	Armed conflict and insecurity	Accessibility of 64 essential medicines on day of survey	WHO/HAI methodology
Alemayehu et al. [28]	Ethiopia (Eastern Amhara)	2023	IDP camp outpatient clinics (11 clinics across five camps)	Internally displaced persons	Armed conflict and insecurity	Facility stock accessibility assessed through observation and staff interviews	Not assessed
Zeru et al. [29]	Ethiopia (Northern Amhara)	2022	Health facilities in war-affected zones (151 facilities)	General population	Armed conflict and insecurity	Direct observation of medicine accessibility using WHO SARA tool	Not assessed
Aljadeeah et al. [30]	Gaza Strip (occupied Palestinian territory)	2025	Public hospitals, humanitarian centers, and private pharmacies (14 facilities)	General population	Armed conflict and insecurity	Facility-level point accessibility of medicines using adapted WHO/HAI method	Not assessed
Deng et al. [31]	South Sudan (Juba County)	2023	Public facilities, private pharmacies, private clinics, and faith-based facilities (55 facilities)	General population	Armed conflict and insecurity	Percentage of facilities with tracer medicines accessible on day of survey (WHO/HAI method)	WHO/HAI methodology
Ahmed et al. [32]	Sudan	2024	Public PHC outlet pharmacies (35 facilities; 975 patients)	General population	Armed conflict and insecurity	Facility accessibility index using WHO/HAI plus patient-reported accessibility	WHO/HAI methodology
Elhassan et al. [33]	Sudan (Kassala State)	2024	Oncology center survey (100 cancer patients)	Cancer patients	Armed conflict and insecurity	Patient-reported accessibility to chemotherapy medicines	Patient-reported out-of-pocket cost per treatment cycle
Rabih et al. [34]	Sudan	2024	Government-run primary healthcare centers (50 facilities)	General population	Armed conflict and insecurity	Direct observation of essential medicine accessibility using Service Delivery Indicator survey	Not assessed
Aljadeeah et al. [8]	Syria (North and East Syria)	2024	Hospitals, primary healthcare centers, and private pharmacies (37 facilities)	Patients with NCDs	Armed conflict and insecurity	Percentage accessibility of medicines using WHO/HAI methodology	Medicine price analysis and sector price comparison
Njuguna et al. [35]	Uganda (North-east)	2022	Public health facilities in drought-affected districts (150 facilities)	General population	Drought and food insecurity emergency	Accessibility of tracer medicines observed in facility stores	Not assessed
Bilousova et al. [36]	Ukraine	2015–2025	Narrative review and pharmacoeconomic analysis of national medicine pricing	Patients with coronary heart disease	Armed conflict and insecurity	Registration and reimbursement coverage of essential medicines	Price variability analysis and out-of-pocket expenditure assessment
Mohamed Ibrahim et al. [10]	Yemen	2017–2018	Public hospitals, private hospitals, private pharmacies, and PHC centers (30 facilities)	General population	Armed conflict and insecurity	Percentage of facilities stocking medicines on day of survey (WHO/HAI method)	Not assessed

**Fig. 2:**
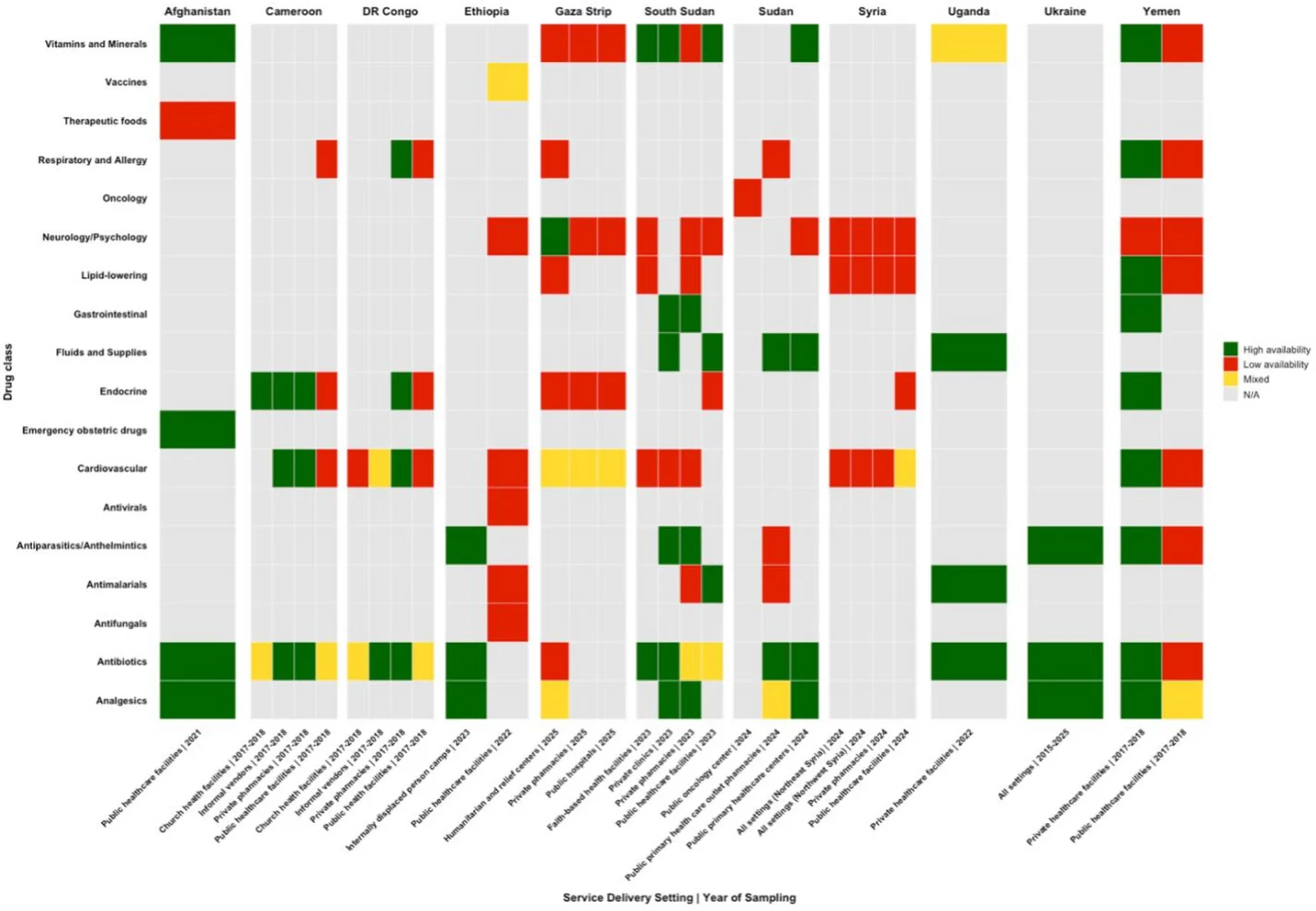
Accessibility of essential medicines categories across countries, health sectors, and facility types

Across the included studies, accessibility was mainly measured using the WHO/HAI methodology, which assesses the percentage of facilities stocking tracer medicines on the day of the survey, as applied in studies from Cameroon, the Democratic Republic of Congo, Chad, South Sudan, Gaza, Syria, Sudan, and Yemen [8, 10, 25–29, 34]. Other measurements included direct observation using the WHO SARA tool in Ethiopia [30], continuous 30-day assessment of medicines accessibility in Afghanistan [31], patient-reported accessibility in Sudan [29], and registration and reimbursement coverage as a proxy for accessibility in Ukraine [32]. Across the included studies, medicine accessibility varied by country and health sector type. Across settings, medicines used for acute infectious conditions—such as antibiotics, analgesics, and oral rehydration salts—were generally more accessible than medicines used for chronic noncommunicable diseases, including antihypertensives, antidiabetic agents, and antiepileptics. Table 2 summarizes the accessibility of medicines across different health sectors and geographical regions in the included studies, while Fig. 2 illustrates accessibility findings. In Sudan, several essential medicines had low accessibility at public primary healthcare facilities—including antiparasitic agents, analgesics, and injectable antimalarials [29, 34]. In contrast, commonly used antibiotics and intravenous fluids showed relatively high accessibility [29, 34]. Severe and prolonged shortages were observed in a public oncology center in Sudan lasting from one month to two years [33]. In South Sudan, both public and private sectors experienced low accessibility of medicines for chronic conditions, including antihypertensives, antidepressants, antidiabetic agents, and pediatric formulations. However, antibiotics, antimalarials, oral rehydration salts (ORS), and micronutrient supplements were generally highly accessible [28]. Similar patterns were observed in Yemen and Syria, where medicines for noncommunicable diseases—particularly cardiovascular, lipid-lowering, and antiepileptic drugs—were reported as having low accessibility across public facilities and private pharmacies [8, 10]. In Ethiopia, gaps in accessibility were noted for selected vaccines, pediatric antiretroviral medicines, antifungals, and rectal artesunate in public healthcare facilities, while routine childhood vaccines remained widely accessible [30]. In internally displaced persons (IDP) camps in Ethiopia, only basic antibiotics and analgesics were reported as accessible [35]. In the occupied Palestinian territory (Gaza Strip), chronic disease medicines and insulin were mostly inaccessible across public hospitals, humanitarian centers, and private pharmacies, while selected cardiovascular medicines and analgesics showed better accessibility [27]. Studies from Cameroon, the Democratic Republic of Congo, and Uganda indicated high accessibility of commonly used antibiotics and antimalarials across most sectors. However, combination antibiotics and medicines for chronic conditions remained inaccessible [25, 36]. In Chad, overall medicine accessibility remained relatively low (below 60%) across both public and private sectors [26]. In Afghanistan, public healthcare facilities reported limited accessibility of therapeutic foods, while basic medicines such as antibiotics, painkillers, and vitamins were more accessible [31]. Across settings, a consistent pattern emerged in which medicines used for acute infectious conditions remained relatively accessible, whereas medicines used for chronic diseases were more frequently inaccessible.

**Table 2:** Accessibility of essential medicines in conflict-affected settings by country and healthcare setting

Country	Sampling Year	Location	Setting	Low accessibility medicines (< 25%)	High accessibility medicines (> 75%)
Sudan [32]^32^	2024	River Nile; Kassala; Gadarif; Blue Nile; North Kordofan	Public primary health care outlet pharmacies	Mebendazole suspension; Mebendazole tablets; Acetylsalicylic acid tablets; Artesunate injection; Benzyl benzoate lotion; Salbutamol tablets	Paracetamol tablets; Amoxicillin tablets/capsules; Metronidazole tablets; Ringer lactate infusion
Sudan [33]	2024	Kassala State (Eastern Sudan)	Public oncology center	Inaccessible for 2 years: Procarbazine capsule/tablet; Pegaspargase injection Inaccessible for 1 year: Melphalan tablet; Trastuzumab injection; Imatinib tablet; Thalidomide capsule Inaccessible for 6 months: Mercaptopurine tablet; Bortezomib injection; Lenalidomide capsule Inaccessible for 4 months: Chlorambucil tablet; Cisplatin injection; Dacarbazine injection; Etoposide injection; Oxaliplatin injection Inaccessible for 2 months: Mesna injection Inaccessible for 1 month: Docetaxel injection	-
Sudan [34]	2024	Red Sea; River Nile; White Nile; Gadarif	Public primary healthcare centers	Promethazine injection	Metronidazole tablet; Paracetamol tablet; Ringer’s lactate infusion; Sodium chloride 0.9% infusion; Dextrose 5% infusion; Injection water injection; Syringes consumable; Amoxicillin capsule; Folic acid tablet
South Sudan [31]	2023	Juba County	Public healthcare facilities	Amitriptyline tablet; Cefixime oral suspension; Co-trimoxazole oral suspension; Diazepam tablet; Metformin tablet	Amoxicillin 250 capsule; Artesunate + amodiaquine adult tablet; Ceftriaxone injection vial; Ciprofloxacin tablet; Doxycycline tablet/capsule; ORS sachet; Vitamin A capsule; Zinc sulphate tablet
			Private pharmacies	Amitriptyline tablet; Artesunate + amodiaquine infant tablet; Atorvastatin tablet; Cefixime oral suspension; Ferrous sulphate + folic acid tablet; Lisinopril tablet	Albendazole chewable tablet; Amoxicillin capsule/tablet; Amoxicillin 250 capsule; Ceftriaxone injection vial; Ciprofloxacin tablet; Diclofenac tablet; Doxycycline tablet/capsule; Metronidazole tablet; Omeprazole capsule
			Private clinics	Lisinopril tablet; Propranolol tablet	Albendazole chewable tablet; Amoxicillin capsule/tablet; Ceftriaxone injection vial; Ciprofloxacin tablet; Diclofenac tablet; Doxycycline tablet/capsule; Ibuprofen tablet; Omeprazole capsule; ORS sachet; Zinc sulphate tablet
			Faith-based health facilities	Amitriptyline tablet; Atorvastatin tablet; Lisinopril tablet; Propranolol tablet	Azithromycin tablet; Ceftriaxone injection vial; Doxycycline tablet/capsule; Metronidazole tablet; Vitamin A capsule; Zinc sulphate tablet
Ethiopia [29]	2022	Amhara region (Northern Ethiopia)	Public healthcare facilities	Measles vaccine; Anti-rabies vaccine; Carbamazepine tablet; Angiotensin II receptor antagonists tablet; Rectal artesunate suppository; Antifungal medicines; Pediatric antiretroviral medicines tablet/syrup	DPT–HiB–HepB vaccine; Polio vaccine; Pneumococcal conjugate vaccine; BCG vaccine
Ethiopia [28]	2023	Eastern Amhara	Internally displaced person camps	-	Paracetamol tablet; Amoxicillin tablet; Albendazole tablet; Cotrimoxazole suspension
Occupied Palestinian territory (Gaza Strip) [30]	2025	Northern Gaza and Southern Gaza	Public hospitals	Metformin tablet; Carbamazepine tablet; Amlodipine tablet; Ferrous salt vial	Enalapril tablet; Losartan tablet; Furosemide tablet; Spironolactone tablet
			Humanitarian and relief centers	Ibuprofen tablet; Diclofenac tablet; Cefixime tablet; Ceftriaxone vial; Insulin Human Mixtard 70/30; Amlodipine tablet; Aspirin tablet; Atorvastatin tablet; Budesonide inhaler; Ferrous salt vial	Paracetamol tablet; Valproic acid tablet; Furosemide tablet; Spironolactone tablet
			Private pharmacies	Metformin tablet; Insulin Human Mixtard 70/30; Carbamazepine tablet; Bisoprolol tablet; Amlodipine tablet; Ferrous salt vial; Levothyroxine tablet	Propranolol tablet
Syria [8]	2024	Northeast Syria	All settings	Lisinopril tablet; Nebivolol tablet; Simvastatin tablet; Oxcarbazepine tablet	-
		Northwest Syria	All settings	Lisinopril tablet; Amlodipine + Telmisartan tablet; Simvastatin tablet; Oxcarbazepine tablet	-
		Northeast and Northwest Syria	Public healthcare facilities	Lisinopril tablet; Amlodipine + Telmisartan tablet; Nebivolol tablet; Losartan + Hydrochlorothiazide tablet; Valsartan + Hydrochlorothiazide tablet; Atorvastatin tablet; Rosuvastatin tablet; Simvastatin tablet; Metformin + Sitagliptin tablet; Carbamazepine tablet; Oxcarbazepine tablet; Levetiracetam tablet	Amlodipine tablet
		Northeast and Northwest Syria	Private pharmacies	Lisinopril tablet; Amlodipine + Telmisartan tablet; Simvastatin tablet; Oxcarbazepine tablet	-
Ukraine [36]	2015–2025	Nationwide	All settings	-	Paracetamol tablet, Amoxicillin tablet, Albendazole tablet, Cotrimoxazole suspension
Yemen [10]	2017–2018	Aden, Lahij, Abyan	Public healthcare facilities	Chlorpheniramine tablet; Phenytoin sodium syrup; Praziquantel tablet; Cloxacillin sodium suspension; Folic acid tablet; Acetylsalicylic acid tablet; Clopidogrel tablet; Glyceryl trinitrate sublingual tablet; Propranolol tablet; Hydrochlorothiazide tablet; Nifedipine capsule/tablet; Chlorpromazine HCl tablet; Salbutamol syrup; Digoxin tablet; Atorvastatin tablet; Simvastatin tablet; Methyldopa tablet	Paracetamol syrup
			Private healthcare facilities	Phenytoin sodium syrup	Ibuprofen tablet; Paracetamol syrup; Chlorpheniramine tablet; Albendazole tablet; Amoxicillin capsule; Erythromycin suspension; Ciprofloxacin tablet; Metronidazole tablet; Folic acid tablet; Acetylsalicylic acid tablet; Nifedipine capsule/tablet; Furosemide tablet; Ranitidine tablet; Glibenclamide tablet; Metformin tablet; Salbutamol syrup; Digoxin tablet; Atorvastatin tablet; Simvastatin tablet; Methyldopa tablet
Uganda [35]	2022	Karamoja and surrounding drought-affected districts	Private healthcare facilities	Iron syrup	Amoxicillin capsule; Vitamin A capsule; Tetracycline/Chloramphenicol capsule; ORS sachet; Zinc tablet; Artemisinin-based combination therapy tablet; Folic acid tablet
Chad [27]	2023	Nationwide	Public healthcare facilities	Overall accessibility: 44.6% in hospitals and health centers; 46.5% in national hospitals; 59.3% in provincial hospitals; 53% in district hospitals; 38.8% in basic health centers	
			Private sector	Overall accessibility: 37.8% in private wholesalers; 47.9% in pharmacies; 58.4% in private depots; 48.4% in contractual sector	
Democratic Republic of Congo [26]	2017–2018	Ituri, North-Kivu, South-Kivu, Tanganyika	Public healthcare facilities	Amoxicillin/clavulanic acid capsule; Atenolol tablet; Hydrochlorothiazide tablet; Metformin tablet; Salbutamol tablet	Amoxicillin capsule; Ciprofloxacin tablet; Doxycycline capsule; Metronidazole tablet; Sulfamethoxazole/trimethoprim tablet
			Church health facilities	Amoxicillin/clavulanic acid tablet/capsule; Atenolol tablet	Amoxicillin capsule; Ciprofloxacin tablet; Metronidazole tablet; Sulfamethoxazole/trimethoprim tablet
			Private pharmacies	-	Amoxicillin capsule; Ciprofloxacin tablet; Doxycycline capsule; Metronidazole tablet; Penicillin V tablet; Sulfamethoxazole/trimethoprim tablet; Furosemide tablet; Metformin tablet; Salbutamol tablet
			Informal vendors	Hydrochlorothiazide tablet	Amoxicillin capsule; Ciprofloxacin tablet; Metronidazole tablet; Penicillin V tablet; Sulfamethoxazole/trimethoprim tablet; Furosemide tablet
Cameroon [26]	2017–2018	Western regions of Cameroon	Public healthcare facilities	Penicillin V tablet; Metformin tablet; Glibenclamide tablet; Hydrochlorothiazide tablet; Furosemide tablet; Salbutamol tablet	Amoxicillin tablet/capsule; Ciprofloxacin tablet; Metronidazole tablet; Sulfamethoxazole/Trimethoprim tablet
			Church health facilities	Penicillin V tablet	Amoxicillin tablet/capsule; Ciprofloxacin tablet; Metronidazole tablet; Doxycycline tablet/capsule; Sulfamethoxazole/Trimethoprim tablet; Metformin tablet
			Private pharmacies	-	Amoxicillin tablet/capsule; Ciprofloxacin tablet; Metronidazole tablet; Doxycycline tablet/capsule; Sulfamethoxazole/Trimethoprim tablet; Furosemide tablet; Hydrochlorothiazide tablet; Metformin tablet; Glibenclamide tablet
			Informal vendors	-	Amoxicillin tablet/capsule; Ciprofloxacin tablet; Metronidazole tablet; Doxycycline tablet/capsule; Sulfamethoxazole/Trimethoprim tablet; Furosemide tablet; Hydrochlorothiazide tablet; Metformin tablet; Glibenclamide tablet
Afghanistan [25]	2021	Nationwide	Public healthcare facilities	Therapeutic foods	Vitamins and supplements; antibiotics; emergency obstetric drugs; general painkillers

**Fig. 3:**
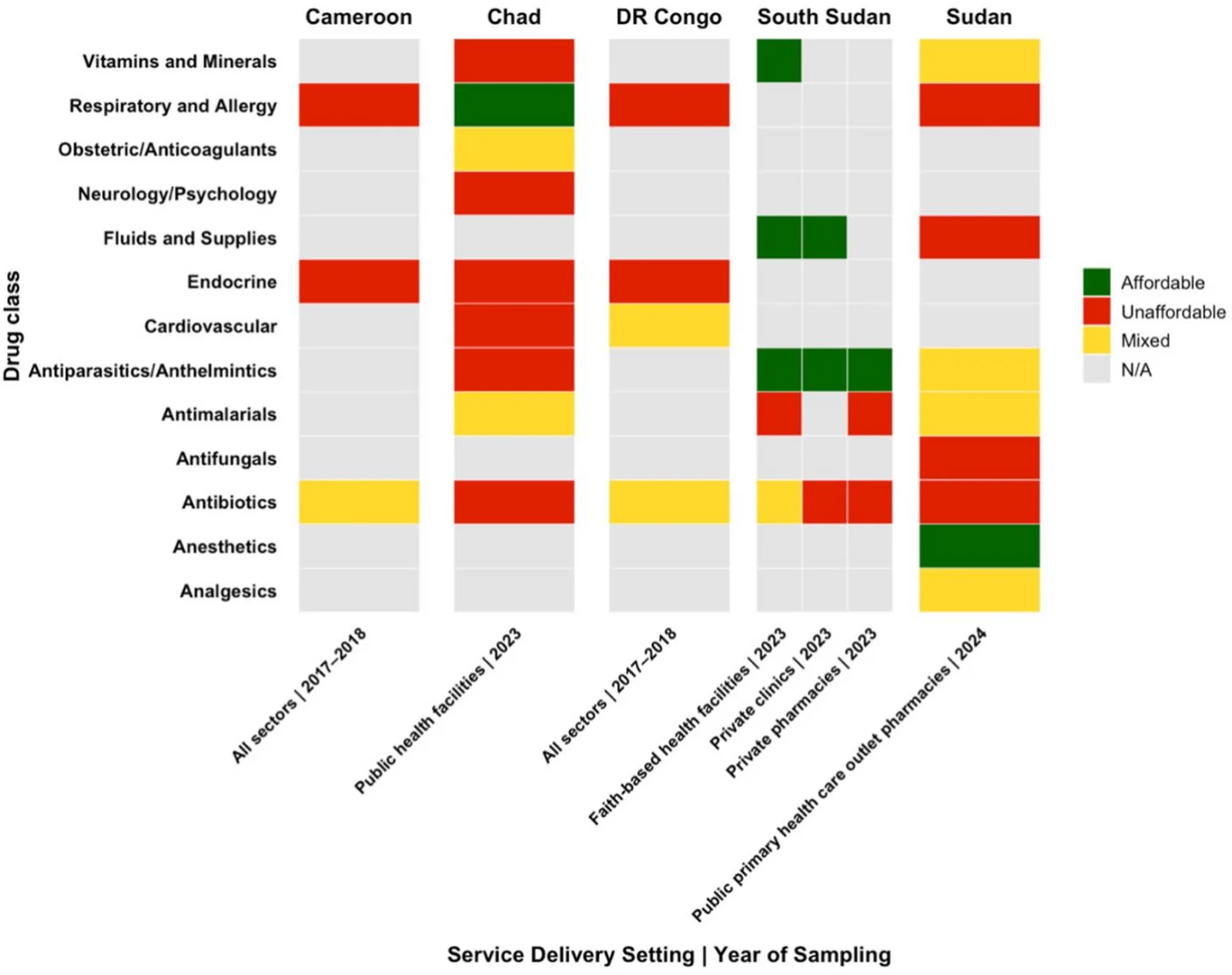
Affordability of essential medicines categories across countries, health sectors, and facility types

Across the included studies, medicine affordability varied by country, sector, and insurance status. Affordability was most commonly assessed using the WHO/HAI method, which measures the number of days’ wages required for the lowest-paid government worker to purchase a course of treatment [25, 26, 28, 29]. Medicines were considered affordable if the cost of treatment required one day’s wage or less [15]. However, some studies used alternative approaches (e.g., price variability analysis [32], medicine prices [8], and out-of-pocket expenditures [33]). Table 3 summarizes the affordability of medicines across different health sectors and geographical regions in the included studies, while Fig. 3 illustrates affordability findings. In Sudan, affordability of essential medicines differed between insured and uninsured patients in public primary healthcare outlet pharmacies. While a limited number of medicines—such as benzyl benzoate lotion and selected analgesics and supplements—were affordable for insured patients, most commonly prescribed medicines, including antibiotics, antiparasitics, iron supplements, bronchodilators, and intravenous fluids, were unaffordable for uninsured patients [29]. In South Sudan, medicines were reported to be provided free of charge in public healthcare facilities. However, affordability challenges were prominent in private pharmacies, private clinics, and faith-based health facilities, where most antibiotics and antimalarial medicines were unaffordable, with only a small number of basic medicines, such as albendazole and ORS remaining affordable [28]. In Chad, artemether–lumefantrine, salbutamol injection, and oxytocin were affordable in public health facilities; however, the majority of essential medicines—particularly antibiotics, insulin, anticoagulants, and medicines for chronic conditions—were unaffordable across both public and private sectors [26]. Similarly, in Cameroon and the Democratic Republic of Congo, commonly used antibiotics were generally affordable across sectors, whereas medicines for noncommunicable diseases, combination antibiotics, and respiratory medicines were frequently reported as unaffordable [25]. 

One study in Syria reported medicine prices based on the selling price to end-users. Median prices varied notably between Northeast and Northwest Syria, and medicines were generally more expensive in Northwest Syria. For cardiovascular medicines, antihypertensives such as captopril and amlodipine were priced at approximately USD 0.53–0.70 in Northeast Syria, compared with higher median prices in Northwest Syria (e.g., USD 1.39 for captopril and USD 0.90 for amlodipine). A similar pattern was observed for diabetes medicines, with metformin, gliclazide, and metformin–sitagliptin combinations priced higher in Northwest Syria, and insulin human mixtard was accessible only in Northwest Syria at USD 3.33 per vial. Epilepsy medicines including carbamazepine, oxcarbazepine, and levetiracetam were among the most expensive medicines in both regions [8]. 

Another study reported the out-of-pocket expenditures incurred per chemotherapy cycle among patients receiving care at a public oncology facility in Sudan. Due to prolonged shortages of essential chemotherapy medicines resulting from conflict-related disruptions to national supply chains, many required drugs were frequently inaccessible at the center and patients had to purchase out-of-stock chemotherapy medicines from private pharmacies. The cost of purchasing chemotherapy drugs from the private pharmacies ranged from USD 23.33 to USD 1,500.00 per chemotherapy cycle, with a mean cost of USD 691.63 ± 367.21 [33].

In Ukraine, affordability of essential medicines for coronary heart disease was assessed through price variability and out-of-pocket expenditure analysis. The study reported substantial price variability across medicines and used liquidity ratios to indicate the differences between minimum and maximum market prices. Atorvastatin and indapamide showed high variability and liquidity ratios exceeding 2. Although most medicines were included in national reimbursement programs, approximately 46.7% of medicines prescribed for long-term cardiovascular treatment were still purchased out-of-pocket by patients [32]. 

**Table 3:** Affordability of essential medicines in conflict-affected settings by country and healthcare setting

Country	Year	Location	Setting	Affordable medicines (≤ 1 day’s wage)	Unaffordable medicines (> 1 day’s wage)
Sudan	2024	River Nile; Kassala; Gadarif; Blue Nile; North Kordofan	Public primary health care outlet pharmacies	Benzyl benzoate lotion (insured & uninsured); Paracetamol tablets (insured only); Lidocaine injection (insured only); Folic acid tablets (insured only); Artemether–lumefantrine tablets (borderline affordability for insured patients only)	Paracetamol tablets (uninsured); Mebendazole tablets; Mebendazole suspension; Acetylsalicylic acid tablets (uninsured); Amoxicillin tablets/capsules; Amoxicillin suspension; Metronidazole tablets; Cotrimoxazole suspension; Ferrous salt tablets; Ferrous salt oral solution; Fusidic acid ointment; Salbutamol tablets; Ciprofloxacin eye drops; Ringer lactate infusion; Artesunate injection (uninsured)
South Sudan [31]	2023	Juba County	Public healthcare facilities	All accessible medicines (free of charge)	-
			Private pharmacies	Albendazole tablet	Amoxicillin capsule; Cefixime suspension; Ciprofloxacin tablet; Azithromycin tablet; Co-trimoxazole suspension; Doxycycline capsule; Sulfadoxine–pyrimethamine
			Private clinics	Albendazole tablet; ORS sachet (borderline)	Amoxicillin (all forms); Cefixime suspension; Ciprofloxacin tablet; Azithromycin tablet; Doxycycline capsule; Co-trimoxazole suspension
			Faith-based health facilities	Albendazole tablet; Metronidazole 200 mg; Zinc sulphate 20 mg; ORS sachet	Amoxicillin (all forms); Cefixime suspension; Ciprofloxacin tablet; Azithromycin; Sulfadoxine–pyrimethamine
Chad [27]	2023	Nationwide	Public health facilities	Artemether–lumefantrine; Salbutamol injection; Oxytocin	Albendazole; Artesunate; Amoxicillin suspension; Ceftriaxone injection vial; Enoxaparin; Iron sulfate + folic acid; Glibenclamide; Mixed insulin; Methyldopa; Phenobarbital
			Private & contractual sector	None reported	Albendazole; Artesunate; Amoxicillin; Ceftriaxone injection vial; Enoxaparin; Iron sulfate + folic acid; Insulin; Methyldopa; Phenobarbital; Salbutamol injection
Cameroon [26]	2017–2018	Western Cameroon	All sectors	Amoxicillin; Ciprofloxacin; Doxycycline; Metronidazole; Sulfamethoxazole/trimethoprim	Amoxicillin/clavulanic acid; Penicillin V; Metformin tablet; Glibenclamide tablet; Salbutamol
DR Congo [26]	2017–2018	Ituri, North-Kivu, South-Kivu, Tanganyika	All sectors	Amoxicillin capsule; Ciprofloxacin tablet; Doxycycline; Metronidazole tablet; Sulfamethoxazole/trimethoprim; Furosemide	Amoxicillin/clavulanic acid capsule; Metformin tablet n; Atenolol tablet; Hydrochlorothiazide tablet; Salbutamol

## Interpretation

Three key patterns emerged from this review. First, medicines used to treat acute infectious conditions were generally more accessible than medicines for chronic noncommunicable diseases. Second, public-sector facilities frequently experienced shortages of essential medicines, while private-sector outlets often had higher accessibility but at higher prices. Third, conflict-related disruptions to supply chains and health system financing appear to exacerbate both accessibility and affordability barriers. Taken together, these findings suggest that conflict does not affect all essential medicines equally; rather, it appears to shift pharmaceutical accessibility toward acute care while leaving chronic disease treatment more vulnerable to disruption.

Barriers to equitable access to essential medicines can be understood through a supply-side and demand-side lens [37]. Accessibility barriers are by nature supply-side, and reflect the health system’s capacity to procure, distribute, and maintain medicines at the facility level [37, 38]. Conflict undermines this capacity through damaged infrastructure, fragmented governance, workforce shortages, and disrupted financing [39]. Affordability barriers, by contrast, can stem from both sides: on the supply side, shortages in public facilities — where medicines would otherwise be free or subsidized — force patients into private markets where the prices are higher; on the demand side, conflict-driven economic shocks, loss of livelihoods, and inflation lower households’ purchasing power [37, 40, 41]. 

Across the included studies, conflict was identified consistently as a barrier to essential medicines accessibility through disruption of pharmaceutical supply chains, especially in public-sector and primary care settings [10, 28–30, 34, 35]. Armed conflict and insecurity disrupt pharmaceutical supply chains via damaged infrastructure, restricted transport, sanctions, and fragmented governance, leading to stock-outs and low facility-level accessibility, as reported in Sudan, Gaza, Yemen, and Northern Ethiopia [10, 27, 29, 30]. Moreover, conflict-driven displacement, workforce shortages, and weakened inventory management exacerbate shortages at service delivery points, as seen in IDP camps in Ethiopia [35]. These issues are compounded by economic shocks—loss of livelihoods, inflation, and rising transport costs—that increase medicine prices and jeopardize medicine affordability [28–33]. Another affordability challenge arises when medicines are not accessible in the public sector, and patients are forced to seek care in the private sector and pay more out of pocket for their medicines that would have been free of charge had they been accessible in public healthcare facilities [33]. 

Many fragile and conflict-affected health systems rely heavily on external financing, including humanitarian and emergency aid, to maintain essential health services and pharmaceutical supply chains [42, 43]. While these mechanisms may temporarily sustain medicine accessibility, they can also create structural vulnerabilities when conflict intensifies [44]. Political instability, sanctions, disrupted banking systems, or shifts in donor priorities may interrupt procurement and delay medicine importation and stock maintenance [45, 46]. Consequently, countries with greater dependence on external funding for health system financing may face amplified challenges in maintaining consistent supplies of essential medicines during periods of acute conflict [47]. These vulnerabilities may further compound the supply chain disruptions and price inflation already associated with armed conflict [48]. Moreover, lower accessibility of medicines used to treat noncommunicable diseases compared to medicines used for infectious conditions likely reflects the structure of humanitarian health responses, which traditionally prioritize emergency care, communicable diseases, and maternal and child health services [49]. This pattern may help explain why medicines for chronic diseases were consistently less accessible across multiple settings captured in this review.

In addition to logistical disruptions, regulatory system challenges may also influence accessibility to essential medicines in conflict settings. Armed conflict can weaken national medicines regulatory authorities, disrupt routine pharmaceutical oversight, and fragment procurement and distribution systems [50]. Weak regulatory oversight may increase the circulation of substandard or falsified medicines and contribute to price volatility in private markets, thereby compounding barriers to medicine accessibility during conflict [51]. 

These findings have implications for both short-term humanitarian response and long-term health system strengthening. In the immediate term, humanitarian actors should ensure that emergency medicine kits and procurement frameworks include NCD medicines alongside medicines for acute infectious conditions, given the consistent finding that chronic disease medicines are disproportionately inaccessible in conflict settings. Establishing contingency procurement mechanisms in conflict settings is a critical short-term priority to mitigate supply chain disruptions. In the longer term, the dependence of fragile and conflict-affected health systems on external financing for medicine procurement represents a structural vulnerability that requires systemic attention. Strengthening domestic pharmaceutical supply chains, building local procurement capacity, and diversifying supply sources can reduce exposure to the disruptions documented across the included studies. Expanding social health insurance coverage is essential to address the affordability barriers identified in this review. Regulatory system strengthening should also be prioritized to prevent the circulation of substandard medicines that may proliferate when oversight is weakened by conflict.

### Limitations

This study has several limitations. First, most studies assessed medicine accessibility and affordability at the facility level and did not capture household-level access barriers such as travel costs, transportation barriers, or informal payments. As a result, the reported accessibility and affordability status may overestimate effective access to medicines from the perspective of patients. Second, the analysis focused on medicines included in national essential medicines lists and did not assess the accessibility of therapeutic alternatives that may have been used in practice. In some settings, substitute medicines outside the defined list may partially mitigate shortages of specific essential medicines, which could lead to underestimation of functional treatment accessibility. The evidence base also lacks studies specifically examining accessibility and affordability of pediatric formulations, maternal health medicines, and mental health medicines in conflict settings. Third, the evidence base remains relatively sparse. Only 14 studies met the inclusion criteria across diverse conflict-affected settings, highlighting the limited availability of empirical research on the accessibility and affordability of essential medicines in conflict settings. Furthermore, publication bias cannot be excluded, as studies in active conflict zones may face significant barriers to data collection and dissemination, potentially underrepresenting the most severely affected settings in the evidence base. Fourth, the limited reporting of urban or rural location and the absence of refugee-specific data across included studies restrict comparisons across these contextual dimensions. Fifth, restricting inclusion to quantitative studies and excluding qualitative evidence limits insight into barriers and contextual determinants not captured through quantitative outcomes. Lastly, the heterogeneity of measurement methods for accessibility and affordability across included studies restricted a formal meta-analysis and limits the comparability of findings across settings and their generalizability.

## Data Availability

The search strategy, extracted study information, and generated and analyzed during this study are included in this published article and its supplementary information files.

## Abbreviations

BCG: Bacille Calmette–Guérin
DPT–HiB–HepB: Diphtheria, Pertussis, Tetanus, Haemophilus influenzae type b, and Hepatitis B vaccine
EML: Essential Medicines List
HAI: Health Action International
IDP: Internally displaced person
JBI: Joanna Briggs Institute
NCD: Noncommunicable disease
NEML: National Essential Medicines List
ORS: Oral rehydration salts
PHC: Primary health care
PRISMA: Preferred Reporting Items for Systematic Reviews and Meta: Analyses
PROSPERO: International Prospective Register of Systematic Reviews
SANRA: Scale for the Assessment of Narrative Review Articles
SARA: Service Availability and Readiness Assessment
WHO: World Health Organization
WHO/HAI: World Health Organization/Health Action International

## References

[1] Haar RJ, Rubenstein LS. Health in fragile and post-conflict states: a review of current understanding and challenges ahead. Med Confl Surviv. 2012;28:289–316. 10.1080/13623699.2012.743311 23421305

[2] Brück T, d’Errico M, Pietrelli R. The effects of violent conflict on household resilience and food security: evidence from the 2014 Gaza conflict. World Dev. 2014;119:203–23.

[3] World Bank Open Data. World Bank Open Data. https://data.worldbank.org (accessed March 6, 2026).

[4] Muriuki J, Hudson D, Fuad S. The impact of conflict on food security: evidence from household data in Ethiopia and Malawi. Agric Food Secur. 2023;12:41.

[5] Alibhai KM, Ziegler BR, Meddings L, Batung E, Luginaah I. Factors impacting antenatal care utilization: a systematic review of 37 fragile and conflict-affected situations. Confl Health. 2022;16:33. 10.1186/s13031-022-00459-9 PMC9188725 35690840

[6] Purgato M, Barbui C. What is the WHO essential medicines list? Epidemiol Psychiatr Sci. 2012;21:343–5. 10.1017/S204579601200039X PMC6998134 22846155

[7] World Health Organization. Selection of essential medicines at country level: using the WHO model list of essential medicines to update a national essential medicines list. Geneva: World Health Organization; 2020.

[8] Aljadeeah S, Tarrafeta B, Fahed S, Kielmann K, Ravinetto R. Access to essential medicines for noncommunicable diseases during conflicts: The case cardiovascular diseases, diabetes and epilepsy in Northern Syria. PLOS Glob Public Health. 2025;5:e0004744. 10.1371/journal.pgph.0004744 PMC12677547 41343509

[9] Marou V, Vardavas CI, Aslanoglou K, et al. The impact of conflict on infectious disease: a systematic literature review. Confl Health. 2024;18:27. 10.1186/s13031-023-00568-z PMC11000310 38584269

[10] Ibrahim MIM, Alshakka M, Al-abd N, Bahattab A, Badulla W. Availability of essential medicines in a country in conflict: a quantitative insight from Yemen. Int J Environ Res Public Health. 2020. 10.3390/ijerph18010175. PMC7795412 33383771

[11] Saeed AA, Zamel AMA, Ahmed AAG, et al. The struggle for medicine: A valid and reliable cross-sectional study on the impact of war on healthcare access and its consequences for displaced Sudanese citizens. PLOS Global Public Health. 2025;5:e0004867. 10.1371/journal.pgph.0004867 PMC12654929 41296819

[12] Adhikari B, Ranabhat K, Khanal P, et al. Procurement process and shortages of essential medicines in public health facilities: A qualitative study from Nepal. PLOS Glob Public Health. 2024;4:e0003128. 10.1371/journal.pgph.0003128 PMC11065305 38696399

[13] Aebischer Perone S, Martinez E, du Mortier S, et al. Non-communicable diseases in humanitarian settings: ten essential questions. Confl Health. 2017;11:17. 10.1186/s13031-017-0119-8 PMC5602789 28932259

[14] Herrera-Ramirez I, Orozco-Nuñez E, Guerra G, Dreser-Mansilla A, Molina-Salazar RE. Access to essential medicines in low- and middle-income countries: a systematic review of barriers and facilitators. Int J Public Health. 2026;71:1608754. 10.3389/ijph.2026.1608754 PMC12877563 41658422

[15] WHO/HAI project on medicine prices and availability. https://www.who.int/teams/health-product-and-policy-standards/medicines-selection-ip-and-affordability/who-hai-project-medicine-prices-and-availability (accessed July 13, 2026).

[16] Al-Worafi YM. Access to Medicines in Developing Countries. In: Al-Worafi YM, editor. Handbook of Medical and Health Sciences in Developing Countries: Education, Practice, and Research. Cham: Springer International Publishing; 2023. pp. 1–33.

[17] Antoñanzas F, Terkola R, Overton PM, Shalet N, Postma M. Defining and measuring the affordability of new medicines: a systematic review. Pharmacoeconomics. 2017;35:777–91. 10.1007/s40273-017-0514-4 28477220

[18] Fragility. accessed July 13, Conflict, and Violence: Country Classifications. World Bank. https://www.worldbank.org/en/topic/fragilityconflictviolence/brief/classification-of-fragile-and-conflict-affected-situations (2026).

[19] Glossary | DataBank. https://databank.worldbank.org/metadataglossary/gender-statistics/country/FCS (accessed July 13, 2026).

[20] Liberati A, Altman DG, Tetzlaff J, et al. The PRISMA Statement for Reporting Systematic Reviews and Meta-Analyses of Studies That Evaluate Health Care Interventions: Explanation and Elaboration. PLoS Med. 2009;6:e1000100. 10.1371/journal.pmed.1000100 PMC2707010 19621070

[21] Barker TH, Hasanoff S, Aromataris E, et al. The revised JBI critical appraisal tool for the assessment of risk of bias for analytical cross-sectional studies. JBI Evid Synth. 2025. 10.11124/JBIES-24-00523. 40521701

[22] Baethge C, Goldbeck-Wood S, Mertens S. SANRA—a scale for the quality assessment of narrative review articles. Res Integr Peer Rev. 2019;4:5. 10.1186/s41073-019-0064-8 PMC6434870 30962953

[23] Covidence. accessed March 6, - Better systematic review management. Covidence. https://www.covidence.org/ (2026).

[24] Ngorsuraches S, Chaiyakan K. Equitable prices of single-source drugs in Thailand. Appl Health Econ Health Policy. 2015;13:389–97. 10.1007/s40258-015-0165-6 25894738

[25] Schäfermann S, Neci R, Ndze EN, Nyaah F, Pondo VB, Heide L. Availability, prices and affordability of selected antibiotics and medicines against non-communicable diseases in western Cameroon and northeast DR Congo. PLoS ONE. 2020;15:e0227515. 10.1371/journal.pone.0227515 PMC6946586 31910444

[26] Ragomzingba FEZ, Haroun SH, Ngabéré C, et al. National availability, prices and affordability of essential medicines: a comprehensive analysis using WHO/HAI methodology in Chad. Pan Afr Med J. 2025;51:72. 10.11604/pamj.2025.51.72.47698 PMC12500885 41064686

[27] Aljadeeah S, Satheesh G, Hafez S, et al. Availability of essential medicines in 14 remaining health facilities in Gaza. Lancet. 2025;406:1465–7. 10.1016/S0140-6736(25)01819-7 41022094

[28] Deng J, Mayai AT, Kayitare E, Ntakirutimana T, Swallehe O, Bizimana T. Assessment of prices, availability and affordability of essential medicines in Juba County, South Sudan. J Pharm Policy Pract. 2023;16:172. 10.1186/s40545-023-00675-5 PMC10757353 38158563

[29] Ahmed ABM, Hemmeda L, Alrawa S, et al. Access to essential medicines during the Sudanese civil war: a cross-sectional study. Int J Equity Health. 2025;24:277. 10.1186/s12939-025-02647-4 PMC12522604 41094447

[30] Zeru T, Agalu A, Balew M et al. Availability of Essential Medicines and Supplies in health facilities in War zones of Amhara region, Northern Ethiopia. *EthJTranSci* 2023; 1. https://ejts.aphi.gov.et/index.php/ejts/article/view/2 (accessed March 9, 2026).

[31] van Gurp M, Alba S, Ammiwala M, et al. The availability of essential medicines in public healthcare facilities in Afghanistan: navigating sociopolitical and geographical challenges. Health Policy Plan. 2025;40:368–79. 10.1093/heapol/czae121 PMC11886856 39697139

[32] Bilousova N, Tkachenko N, Kozhuharyova N, Dolzhenko M. Research of affordability to essential medicines for coronary heart disease in Ukraine. J Pharm Policy Pract. 2025;18:2470841. 10.1080/20523211.2025.2470841 PMC11921159 40109502

[33] Elhassan MMA, Mohammed AAA, Osman Muhyeldeen FK, et al. Cancer treatment amid armed conflict: Challenges and urgent needs in Sudan. J Cancer Policy. 2025;45:100604. 10.1016/j.jcpo.2025.100604 40466808

[34] Rabih M, Ahmed ABM, Abdelgyoum HA, et al. Availability and readiness of healthcare services at primary healthcare centers during the Sudan war: a cross-sectional study. BMC Prim Care 2025;26:317 10.1186/s12875-025-03015-5 PMC12533350 41102632

[35] Alemayehu TT, Woldeyohanins AE, Gebremeskel ED, et al. Availability of essential medicines, inventory management practices, and related challenges in internally displaced persons (IDP) camps, eastern Amhara, Ethiopia: an explanatory sequential mixed study. Confl Health. 2025;19:56. 10.1186/s13031-025-00697-7 PMC12323210 40760672

[36] Njuguna C, Tola HH, Maina BN, et al. Essential health services delivery and quality improvement actions under drought and food insecurity emergency in north-east Uganda. BMC Health Serv Res. 2023;23:1387. 10.1186/s12913-023-10377-y PMC10714455 38082433

[37] Bigdeli M, Jacobs B, Tomson G, et al. Access to medicines from a health system perspective. Health Policy Plan. 2013;28:692–704. 10.1093/heapol/czs108 PMC3794462 23174879

[38] Mustafa A, Yasmeen A. Access to medicine. Int J Pharm Integr Health Sci. 2022;3:118–21.

[39] Haar R, Rayes D, Tappis H, et al. The cascading impacts of attacks on health in Syria: A qualitative study of health system and community impacts. PLOS Glob Public Health. 2024;4:e0002967. 10.1371/journal.pgph.0002967 PMC11175436 38870115

[40] National Academies of Sciences, Division E H and, Services M, on HC B et al. Factors Influencing Affordability. In: Making Medicines Affordable: A National Imperative. National Academies Press (US), 2017. https://www.ncbi.nlm.nih.gov/books/NBK493090/ (accessed July 13, 2026). 29620830

[41] Belayneh A, Getachew M, Abebe D, et al. Availability and affordability of essential medicines during armed conflict in public hospitals of east Gojjam zone, Amhara region, Ethiopia: a mixed-method institutional-based cross-sectional study. Sci Afr. 2026;31:e03227.

[42] Alani AH, Miller L, Darji B, et al. Assessment of the WHO non-communicable diseases kit for humanitarian emergencies in South Sudan: a retrospective, prospective, observational study. Confl Health. 2023;17:27. 10.1186/s13031-023-00525-w PMC10241119 37277827

[43] Hussein SA, Ali AA, Osman MM, et al. Prolonged multisectoral aid-driven reliance on health systems and governance post-conflict era in Somalia. Trop Med Health. 2026;54:10. 10.1186/s41182-025-00861-y PMC12781686 41508251

[44] HeRAMS Health systems’ reliance on external aid. an assessment of vaccination services. https://www.who.int/publications/m/item/herams-health-systems-reliance-on-external-aid-vaccination-services-2025-07 (accessed March 7, 2026).

[45] Huffstetler HE, Bandara S, Bharali I, et al. The impacts of donor transitions on health systems in middle-income countries: a scoping review. Health Policy Plan. 2022;37:1188–202. 10.1093/heapol/czac063 PMC9558870 35904274

[46] Hosseini SA. Impact of sanctions on procurement of medicine and medical devices in Iran; a technical response. Arch Iran Med. 2013;16:736–8. 24329148

[47] Verjee A, Galad TH, Chang R-Y. Health care without medicine: the impact of war on Sudan’s pharmaceutical manufacturing and supply. Confl Health. 2025;19:28. 10.1186/s13031-025-00667-z PMC12063387 40346623

[48] Bertone MP, Jowett M, Dale E, Witter S. Health financing in fragile and conflict-affected settings: what do we know, seven years on? Soc Sci Med. 2019;232:209–19. 10.1016/j.socscimed.2019.04.019 31102931

[49] Aljadeeah S, Sy H, Michielsen J, et al. Access to medicines and continuity of non-communicable diseases care for forcibly displaced populations: a call for rights-based, comprehensive responses. BMC Glob Public Health. 2025;3:49. 10.1186/s44263-025-00172-8 PMC12125717 40448210

[50] Coulibaly I, Diarra Y, Ahmed MAA, Ravinetto R, Doumbia S, Kielmann K. Contextual constraints and dilemmas influencing health providers’ prescription practices in a conflict-affected area: Qualitative insights from Mopti, Mali. PLOS Glob Public Health. 2025;5:e0004621. 10.1371/journal.pgph.0004621 PMC12530568 41100543

[51] Buckley GJ, Gostin LO, Committee on Understanding the Global Public Health Implications of, Substandard F, Health B, on G, Medicine I. of. Causes of Falsified and Substandard Drugs. In: Countering the Problem of Falsified and Substandard Drugs. National Academies Press (US), 2013. https://www.ncbi.nlm.nih.gov/books/NBK202531/ (accessed March 7, 2026). 24872973

